# Negative Priming Under Rapid Serial Visual Presentation

**DOI:** 10.1371/journal.pone.0037023

**Published:** 2012-05-21

**Authors:** Kin Fai Ellick Wong

**Affiliations:** Department of Management, Hong Kong University of Science and Technology, Hong Kong SAR, China; National Institute of Mental Health, United States of America

## Abstract

Negative priming (NP) was examined under a new paradigm wherein a target and distractors were temporally separated using rapid serial visual presentation (RSVP). The results from the two experiments revealed that (a) NP was robust under RSVP, such that the responses to a target were slower when the target served as a distractor in a previous trial than when it did not; (b) NP was found regardless of whether the distractors appeared before or after the targets; and (c) NP was stronger when the distractor was more distinctive. These findings are generally similar to those on NP in the spatial search task. The implications for the processes causing NP under RSVP are discussed in the current paper.

## Introduction

If a visual stimulus is deliberately ignored in a prime trial as a distractor (i.e., non-targets that people have to ignore and the presence of which may interfere with people's responses to the targets), the response time to the same (or similar) stimulus is often slower in a subsequent probe trial than if an unrelated stimulus was the previous distractor [1]–[3]. This effect is called negative priming (NP) [4]–[5]. NP has been an important effect for researchers to understand the psychological mechanism underlying target selection in selective attention [1]–[5].

Previous research has primarily demonstrated this effect in a context where targets and distractors are simultaneously presented (e.g., they are spatially separated and/or with different colors; i.e., spatial NP). The major objective of the present study is to extend NP research by examining whether NP exists in a two-trial paradigm (i.e., a prime trial followed by a probe trial) resembling spatial NP, except that the targets and distractors are temporally separated under rapid serial visual presentation (RSVP). This effect is called NP under RSVP in this paper.

In RSVP, the stimuli are sequentially presented at the same location on a computer monitor (i.e., a stimulus presented later masks a stimulus presented earlier) with a very fast presentation rate (e.g., 100 ms per item). RSVP has been a major methodology for studying temporal attention [6]–[10]. Extant explanations of spatial NP do not explicitly deal with the temporal dimension of target selection. Studying NP under RSVP, therefore, offers the opportunity to understand how target selection is accomplished when the target and distractors are temporally separated. The present research study seeks to establish how the extant explanations of spatial NP are related to and responsible for NP under RSVP.

This research attempts to advance the existing body of knowledge in two ways. First, a clear demonstration of NP under RSVP will add new empirical constraints that may shed light on the two major theoretical accounts of NP, namely, the selective inhibition-account [1], [2], [11] and the episodic retrieval account [12]–[14]. For example, inhibition-based accounts may need to add an assumption that distractor inhibition occurs very early and quickly. Similarly, retrieval-based accounts may need to add an assumption that encoding episodic information on ignored items can be completed within a very short period of time. The theoretical implications of these empirical constraints will be elaborated in General Discussion.

Second, the two experiments allow a comparison that is useful in evaluating the extent to which extant explanations are responsible for the occurrence of NP under RSVP. The two experiments in the present research differed in terms of whether the distractor letter or the target letter was distinctively different from the remaining items in an RSVP list. The target letter was presented in red, whereas the other items were presented in black in Experiment 1. Thus, NP was observed when a non-distinctive distractor in a prime trial became a distinctive target in the subsequent probe trial (i.e., a nondistinctive-to-distinctive transition). The distractor letters were presented in red, whereas the other items including the target were presented in black in Experiment 2. Thus, NP was observed when a distinctive distractor in the prime trial became a non-distinctive target in the probe trial (i.e., a distinctive-to-nondistinctive transition). The difference between the two experiments is referred to as “distractor-to-target distinctiveness transition.”

To forecast, NP under the nondistinctive-to-distinctive transition (Experiment 1) was stronger than NP under the distinctive-to-nondistinctive transition (Experiment 2). These findings suggest that the mechanisms posited by the feature mismatch [15], [16] and temporal discrimination accounts [17] are unlikely to be responsible for NP under RSVP because the degree of the feature mismatch and the degree of the new-old discriminability of an item are logically independent of the distractor-target distinctiveness transition. However, these findings can be more naturally explained by the selective inhibition and episodic retrieval accounts. This implication will be discussed in detail in the General Discussion.

I first review four published studies suggesting that NP under RSVP may possibly occur [18]–[21]. I then report on two experiments demonstrating NP under RSVP. More in-depth discussions on the two aforementioned contributions will be stated in the General Discussion.

### Preliminary Evidence for NP under RSVP

Unlike the typical procedures employed in NP research, wherein participants are required to respond to only one target in a trial, the three published studies [18], [19], [21] showing preliminary support for NP under RSVP required the participants to respond to two targets in the same RSVP trial. In addition, the preliminary evidence for the findings under RSVP was observed by Milliken et al. [17] under a non-RSVP task. However, all these studies have limitations that prohibit a clear demonstration of NP under RSVP. These limitations include the (a) reliability of the experimental task, (b) use of different dependent variables, and (c) different selective attention requirements. The preliminary evidence, along with its limitations, is discussed in detail in the succeeding sections.

#### Limitation 1: Reliability of the experimental tasks

In Loach and Mari-Beffa's Experiment 1 [18], target 1 (T1) was defined in terms of color (i.e., red letter), whereas target 2 (T2) was defined in terms of temporal order (i.e., the final letter in RSVP). The participants performed speeded identification to T2 immediately after the end of an RSVP list and reported the identity of T1. In some trials, the distractors following the red target were repeated as the final letter, whereas in the other trials, no repetition was made. When the distractor was presented 90 ms to 270 ms after T1, the responses to T2 were slower in the distractor repeated condition than in the distractor unrepeated condition. Harris et al.' Experiment 1 [19] observed similar findings when object pictures were presented under RSVP. In this experiment, the participants' task was to report the two targets (defined by color) in each RSVP list. The results revealed that the performance for T2 was impaired when T1 was preceded by a distractor object identical to T2.

Other experiments that applied procedures similar to those adopted by Loach and Mari-Beffa [18] and Harris et al. [19] obtained mixed results. Maki, Frigen, and Paulson [21] observed positive priming in a design similar to that of Loach and Mari-Beffa [18], except that the distractors were strong associates of T2s and that no speeded responses to T2 were required. Loach and Mari-Beffa [18] did not discuss why the difference in procedure yielded inconsistent results. In the same experiment observing NP, Harris et al.' Experiment1 [19] observed positive priming when the orientation of the distractor object was rotated by 90°. The subsequent experiment (Experiment 2) showed no priming for an identical (unrotated) distractor. Harris et al. attribute the lack of NP to the condition that the two experiments presenting distractors at different orientations, thus inducing different levels of suppression:

“In Experiment 1, upright targets occurred amongst upright distractors and so distractors would have had to be strongly suppressed… In contrast, in Experiment 2 an upright T1 was surrounded by rotated distractors … and thus reduced the need to suppress the distractors” (p. 1601).

However, the above attribution was not supported in their subsequent Experiment 4, which showed no priming when all RSVP items were presented in an upright orientation. The authors did not offer further explanations for this null finding. They concluded that their research “yielded no priming (*in Experiments 2 and 4*), and sometimes even negative priming (*in Experiment 1*)” (p. 1065)

One possibility behind the inconsistent NP findings is that the tasks require participants to register more than one target per RSVP trial. As suggested by the attentional blink (AB) literature [7], [8], [10], the requirement for identifying multiple targets in the same RSVP trial likely involves multiple mental operations that could be very sensitive to subtle experimental demands and the nature of the materials. Harris et al. [19] acknowledged a similar idea in explaining why NP could not be consistently observed in their study, proposing that the tasks in their experiments involved multiple mechanisms. They interpreted that the null NP effects were found because positive priming effects due to repetition (i.e., repetition priming) was cancelled by the NP effects due to suppression:

“the lack of priming in the same-orientation PD condition is because of a combination of positive priming mediated by orientation-invariant stimulus attributes … and inhibition at the perceptual or view-specific level as a result of distractor suppression” (p. 1601).

In summary, the evidence of NP under RSVP from Loach and Mari-Beffa [18] and Harris et al. [19] is suggestive, but not conclusive. In particular, the inconsistent findings from the experiments using similar procedures and manipulations suggest that these experimental tasks might not be sufficiently reliable to induce consistent findings on NP. For example, Harris et al. [19] proposed that their tasks might have induced both positive priming and NP effects simultaneously, resulting in no priming because the two effects cancelled each other out. Therefore, a reliable NP effect under RSVP is predicted when only one target per trial exits.

The present research addresses this limitation by having only one target per RSVP list. It is important to note that the above reasoning does not imply that NP does not occur in dual- or multiple-target RSVP tasks, which are typically used in AB research. The key notion is that responses to the target in dual- or multiple-target RSVP task (vs. single-target RSVP) are more likely to be determined by mechanisms other than the ones responsible for NP because of the involvement of more sophisticated and complicated mechanisms. Thus, the single-target RSVP procedure appears to be a better paradigm because it appears to yielded less nosier responses. Furthermore, the use of a single-target RSVP allows direct comparisons between past NP findings and the current findings because typical NP studies involve a single target per trial.

#### Limitation 2: Use of different dependent variables

Kihara et al. [20] more recently found a distractor devaluation effect on the distractors presented in the dual-target RSVP task. This effect refers to the findings that people gave a lower favorability rating to nonsense stimuli that were previously ignored in RSVP than to novel stimuli. Their study was conducted in two phases. In the first phase, the participants performed a standard dual-target RSVP task that induced AB, in which they were required to report the two targets embedded with a series of distractors in RSVP. In the second phase, the participants gave favorability ratings to items that were distractors in the previous RSVP phase and to novel items. The participants gave lower ratings to the distractor items than to the novel items (i.e., the distractor devaluation effect) (a) when the distractors were presented after T1, but not before T1; and (b) when AB occurred as indicated by T2 that was not identified. On the basis of these findings, Kihara et al. [20] interpreted that distractors are inhibited during AB.

These findings suggest the existence of NP under RSVP. That is, the temporally presented distractors were first inhibited in RSPV. In the subsequent evaluation, when the distarctors became the target for evaluation, people gave a more negative rating that is presumably because of the carryover effect of the inhibition.

Although the distractor devaluation effect found in RSVP by Kihara et al. [20] suggests the existence of NP under RSVP, two issues remain to be addressed. First, similar to Limitation 1 mentioned above, the effect was found only during AB (i.e., for distractors between T1 and T2) with dual-target RSVP, whereas typical NP occurs in a condition where AB does not occur (i.e., a single-target condition). Thus, the distractor devaluation effect may not be found in single-target conditions.

Second, and more important, the dependent variable showing the distractor devaluation effect differs from the typical online dependent variables showing NP (i.e., speed and accuracy). Literature on rating judgment [22], [23] has indicated that making an evaluation rating requires substantial offline mental operations such as memory (e.g., judging information from memory), scaling and weighing (e.g., weighing and mapping the information to an internal scale), and response operations (e.g., preparing actual responses for the ratings). The critical concern in the use of offline dependent variables to capture online mental operations is that it is very difficult to interpret whether the observed effect from the offline measures is due to such online operations as attentional and perceptual processing or to such offline/post-attentional operations as memory, scaling, and response processing.

For example, repetition blindness is an attention phenomenon wherein when two items are presented briefly and consecutively, people have more difficulty in recognizing the second item when the two items are identical than when they are not identical [24]–[26]. This effect was initially observed from offline measures of recall performance of items in RSVP lists. However, the reliance on recall performance to manifest repetition blindness gave rise to a criticism that repetition blindness is a kind of post-attentional failure in reporting information from memory [27], [28] or a memory misattribution problem [29], [30]. The difficulty in concluding that repetition blindness involves an attentional problem was later attenuated when the effect was manifested by the data on online response time to the RSVP items [31], [32]. Similarly, researchers examining online cognitive processing in text comprehension (e.g., phonological activation, lexical access, syntactic integration, etc.) have avoided using offline methods such as ratings and recalling text contents, and instead have relied on online methods such as mouse tracking moving window reading [33], [34] and eye-tracking techniques [35], [36].

This concern on the use of offline measures to study online cognitive processes has led researchers to advocate online methods that can partial out offline mental operations [33]–[36]. Furthermore, this concern and suggestion seem to be particularly salient to the use of ratings to indicate attentional processes because research has shown that ratings are highly vulnerable to post-attentional factors such as scaling factors [23], [37]–[39] and motivational adjustments on rating responses [40], [41]. Therefore, although the use of evaluation ratings might be suggestive of the inhibition in attention, making an inference from this method might not be as conclusive as using other online methods. More direct evidence of NP under RSVP parallel to spatial NP with response times and error rates as dependent variables is needed. To address this limitation, NP under RSVP in the present research was observed in the response time data.

#### Limitation 3: Different selective attention requirements

Milliken et al. [17] observed NP in a procedure similar to, yet distinct from, RSVP. In one of their experiments (Experiment 2) a briefly presented word (i.e., 33 ms), which was out of conscious awareness, was preceded by a 500 ms premask and was followed by a 500 ms postmask. Afterward, two words were displayed in different colors. The participants named the target color word as quickly and as accurately as possible. Their responses were longer when the prime word was the same as the target word than when the words were different. The NP effect disappeared when the target was not presented with a distractor (Experiment 3). In addition, when the exposure duration of the prime was extended to be long enough for conscious awareness (i.e., 200 ms), NP was found when the participants were asked to ignore the prime.

Although the findings from Milliken et al. [17] suggest that a temporally presented prime can induce NP, the procedure they used may not fully capture the temporal characteristics exhibited by the RSVP procedure. There are two differences between the two tasks that make it difficult to conclude that NP under RSVP was clearly observed in Milliken et al. [17]. First, the participants in Milliken et al. were not required to select a target among a series of temporally separated distractors, whereas RSVP explicitly requires the participants to have a much heavier and more direct involvement in the selection of the temporally presented stimuli. Second, the target temporal position in Milliken et al. was fixed such that the participants could simply rely on the temporal cue (without doing target-distractor discrimination) to allocate their attention to the target (e.g., focusing their attention 1 s after the fixation), whereas the target temporal position in RSVP is varied and uncertain.

These two task features in Milliken et al. leads to a concern that the participants might not be actively engaged in the selection during the stimulus was presented because they could identify the target from the temporal cue under a simple selection task. To address this limitation, the tasks in Experiments 1 and 2 (a) required the participants to select a target among temporally separated distractors under RSVP and (b) varied the temporal position of the target in each RSVP trial so that participants could not make the selection by relying only on the temporal cue.

In summary, although the studies by Loach and Mari-Beffa [18] and Harris et al's Experiment 1[19] have suggested that temporal distractor may lead to NP under RSVP, no conclusive evidence is available because other experiments using similar procedures have found either the opposite effect or none at all [19], [21]. Kihara et al. [20] illustrated the distractor negative effects on the favorability ratings in the dual-target RSVP. However, how these findings can be generalized to a single target with speed and accuracy as dependent variables is unclear. Milliken elt al. [17] found NP that was induced by temporally presented prime. However, the procedure in their research may not fully capture selective attention under RSVP.

### The Present Study

The goal of the present research is to offer more direct and clearer evidence of NP under RSVP by addressing the limitations mentioned above. First, to address the limitation that the previous tasks having more than one target per RSVP trial might not be able to yield reliable NP, there was only one target per RSVP list in the present research. Second, to address the limitation on the use of different dependent variables to represent NP, direct evidence of NP under RSVP was observed in the response time data, parallel to the standard manifestation of NP in the literature. Third, to address the limitation on the use of tasks that do not correspond to the selective attention requirement under RSVP, the participants in the present research were required to select a target among temporally separated distractors under RSVP, with the target temporal positions being unpredictable to the participants.

**Figure 1:**
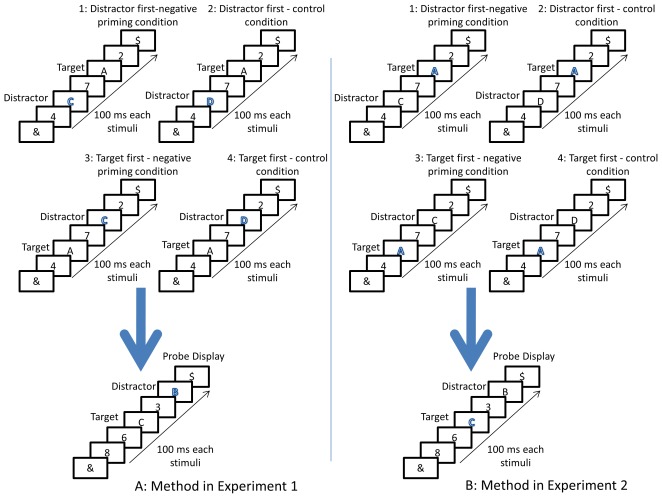
Sample displays in Experiment 1 (A) and Experiment 2 (B). In all cases, a prime display occurred before a probe display. Prime displays were manipulated to have four versions following a 2 (priming condition: NP vs. control) by 2 (display sequence: distractor-first vs. target-first) factorial within-participant design. In the NP condition, the distractor letter in the prime display (“C”) became the target letter in the probe display. In the control condition, the target letter in the probe display was not related to the target letter or distractor letter in the prime display. In the distractor-first condition, the distractor letter occurred before the target letter. In the target-first condition, the target occurred before the distractor letter. In Experiment 1, the distractor letters were in red, whereas the target letters and all other digits were in black. In Experiment 2, the target letters were in red, whereas the distractor letters and all other digits were in black. In both experiments, the display sequence for both target and distractor in the probe display was counterbalanced.

Specifically, the present research modified the traditional two-trial single-target NP paradigm in RSVP to demonstrate NP when the targets and distractors were temporally separated. The unit of analysis showing NP comprised a prime trial, followed by a probe trial. Each trial involved a list of five items presented in RSVP, with each item having 100 ms of exposure time at the same location on a computer display (Figure 1). Each list included three digits and two uppercase letters. The two letters were randomly selected from A, B, C, and D, with no repetition of the two letters and the digits. One letter was presented in bright red, whereas the other letters and all the digits were presented in black.

In Experiment 1, the black letter served as the target letter, whereas the red letter served as the distractor. The two letters were always intervened by a digit. The first letter randomly appeared in Position 1, Position 2, or Position 3 in a temporal sequence. The task of the participants was to identify the target (black) letter as quickly and as accurately as possible while ignoring the distractor (red) letter.

In Experiment 2, NP under RSVP was further examined under an extreme condition in which the distractors were sufficiently distinct from the target. Target distinctiveness was increased by making the red letter as the target and the black letter, and all the other digits (in black) as the distractors. Experiment 2 is essentially a feature search task, in which the goal of the participants was to identify the one with the red color while filtering out the non-red distractors. This experiment effectively tested the robustness of NP under RSVP by allowing the participants to complete the task and ignore the distractor identities.

## Experiment 1: NP under RSVP with Distinctive Distractors

### Methods

#### Participants

Twenty undergraduate students from Hong Kong University of Science and Technology participated in the first experiment. All had normal or corrected-to-normal vision. Each participant completed 128 pairs of trials. Each pair comprised a prime and a probe display.

#### Design, materials, and procedure

For each pair, the probe trial always appeared immediately following the prime trial. Each trial involved a presentation list with three digits and two uppercase letters. The digits were randomly selected from 0 to 9 whereas the letters were either A, B, C, or D. The two letters were always separated by a digit, with a presentation rate of 100 ms per item. The first letter randomly appeared in either the first, second, or third position on the list. One of the letters was presented in red, serving as the distractor. The target letter and all the digits were presented in black. The use of the four-letter identification task followed the previous research on spatial NP [3], [12]. The 100 ms presentation rate followed the previous research on AB [10].

In half of the probe trials, the target was the distractor of the preceding prime display (i.e., the NP condition) or was different from the letters in the prime displays (i.e., the control condition). In half of the prime trials, the distractor appeared before the target (i.e., the prime-distractor-first condition) or the target appeared before the distractor (i.e., the prime-target-first condition). There were two within-subject factors: the priming condition (NP vs. control) and the prime trial sequence (distractor-first vs. target-first). The probe trial sequence was counterbalanced, such that in half of the probe trials, the distractor appeared before the target or the target appeared before the distractor. This counterbalance was conducted to equate the probability of a probe trial sequence. All the subsequent analyses collapsed the data across these two conditions because this manipulation neither interacted with NP in all the analyses nor was it relevant to the research question.

The participants were informed that their task was to respond to the black letter in each trial as quickly and as accurately as possible. The middle and index fingers of the left hand were used to press keys *F* and *V* on a standard QWERTY keyboard. The middle and index fingers of the right hand were used to press keys *J* and *N*. Keys *F*, *V*, *J*, and *N* correspond to letters *A*, *B*, *C*, and *D*, respectively. Each unit of the display comprised a prime trial, followed by a probe trial. Each trial started with a fixation asterisk “&” shown at the center of the monitor for 1,000 ms. Immediately after its offset, five items were presented successively in the same location for 100 ms, each without any interstimulus intervals. The list ended with another asterisk “$” for 100 ms. The next trial began automatically after a response was made, or 3000 ms after the ending asterisk. Eight practice trials were performed at the beginning of the experiment. The feedback information on the accuracy and response time was given in the practice trials after their responses but not in the real experiment. The participants sat about 50 cm away from the computer screen. All stimuli were presented against a light gray background, and were subtended approximately at 0.4°×0.6° of the visual angle.

#### Ethics

I declare that individual participants in the current study gave their written informed consent. The Institutional Review Boards at the Hong Kong University of Science and Technology approved the study.

### Results and Discussion

The trials in which the participants reported the identity of the distractor in the prime displays were removed from the analyses because they did not successfully inhibit the distractor. The removed trials constituted 30.9% of the total trials.

**Figure 2:**
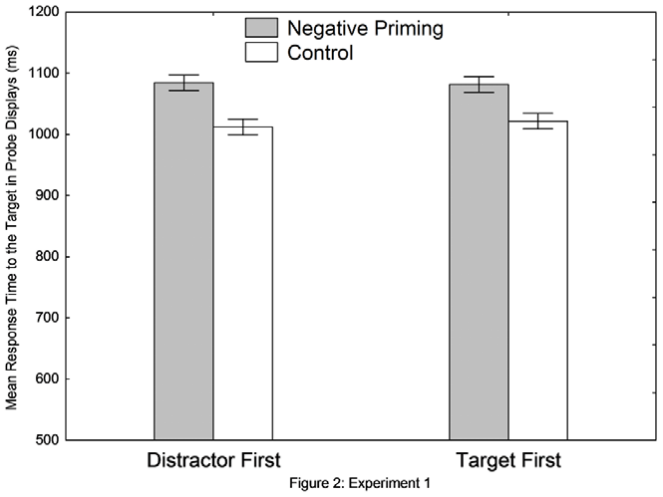
Results of Experiment 1. Vertical bars denote 0.95 confidence intervals (within-participants).

Figure 2 shows the reaction time data as a function of the priming condition (NP vs. control) and the prime trial sequence (distractor-first vs. target-first). A two-way ANOVA revealed a significant main effect of the priming condition, *F* (1, 19) = 8.15, *MSE* = 747.44, *p*<.01, η_p_^2^ = .30. The responses in the NP condition (1083 ms) were significantly longer than those in the control condition (1017 ms). The two-way interaction was not significant, *F*<1, indicating that the 73 ms of NP effect in the distractor-first condition (1,085 ms vs. 1,012 ms) was statistically comparable with the 60 ms NP effect in the target-first condition (1082 ms vs. 1022 ms). No significant results were found in the error rate data.

An additional analysis was conducted to examine whether NP was found in the 30.9% of the removed “capture” trails. The rationale of this analysis is that if a successful target selection in RSVP involves successful processes on distractors (e.g., inhibition or episodic encoding), then NP should not be observed when the distractors are not successfully ignored. The removed trials were the clear cases in which the distractors were not successfully ignored because participants could report their identities. Therefore, NP should not be observed in these trials. Consistent with this rationale, the analysis showed that the mean response time to the probe targets in the NP condition (1052 ms) was statistically comparable with that in the control condition (1112 ms), *F* (1, 19) = 1.38, *p*>.25, and this difference was not statistically dependent on prime trial display sequence, *F*<1.

Experiment 1 showed that (a) NP under RSVP was similar to spatial NP, and that (b) the inhibition starts early and nonselectively for distractors that occur before the target because the inhibition was not dependent on the display sequence; the inhibition in the distractor-first condition (73 ms) was comparable to that in the target-first condition (60 ms).

## Experiment 2: NP under RSVP with a Distinctive Target

### Objectives

Although NP under RSVP was found in Experiment 1, there was one feature of the task that was not completely comparable to the conventional spatial NP task. In Experiment 1, the target letter and the filler digits in the RSVP list were presented in the same color (i.e., black), whereas the distractor letter was presented in a distinctive color (i.e., red). The participants had to differentiate the target letter from the filler digits by their identities. This feature might encourage the participants to adopt a strategy of first processing the identities of all stimuli, followed by processing their colors, which would result in them not ignoring the distractor letter identity. Therefore, distractor letter identities might not be completely irrelevant in Experiment 1. However, the distractor identities were not encouraged to be processed in the typical spatial NP task (e.g., target letter was flanked by the distractor letters [3], [12]).

In Experiment 2, the robustness of NP under RSVP was examined under a condition in which the participants were encouraged to start the selection by attending to the features of the targets while ignoring the identities of the distractors. Target distinctiveness was increased by making the red letter the target and the black letter and all other digits (in black) the distractors. The goal of the participants in this task was to identify the one with the red feature. An effective strategy, therefore, was to pay attention primarily to the color while ignoring the identity before the color was matched. This strategy actually discourages people to process (including to inhibit) the identity of the distractors. Thus, this experiment serves as a strong test of the robustness of NP under RSVP.

### Methods

#### Participants

Twenty-five undergraduate students from Hong Kong University of Science and Technology participated in this experiment. All had normal or corrected-to-normal vision.

#### Design, materials, and procedure

All aspects were identical to those in Experiment 1, except that in each trial, the target letter was always in red, whereas the distractor letter was always in black. The task of the participants was to identify the black letter as quickly and as accurately as possible.

#### Ethics

I declare that the individual participants in the present study gave their written informed consent. The Institutional Review Boards at the Hong Kong University of Science and Technology approved the study.

### Results and Discussion

**Figure 3:**
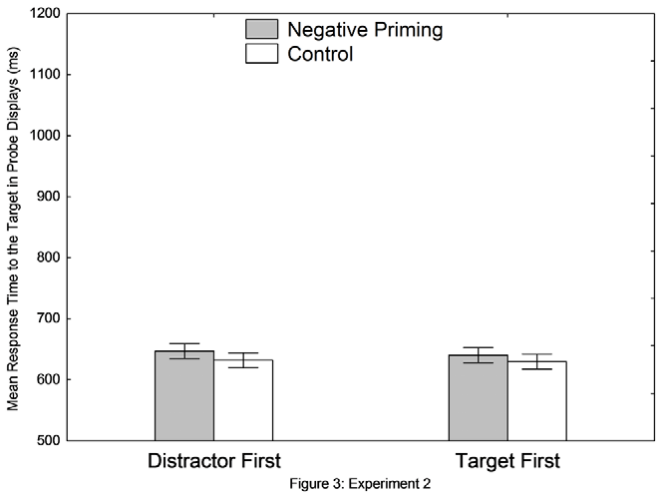
Results of Experiment 2. Vertical bars denote 0.95 confidence intervals (within-participants).

In the analyses, the trials in which the participants reported the identity of the distractor in the prime displays were removed because they did not successfully inhibit the distractor. The removed trials constituted 3.6% of the total trials. Figure 3 shows the reaction time data as a function of priming condition (NP vs. control) and prime trial sequence (distractor-first vs. target-first). Two-way ANOVA revealed a significant main effect of the priming condition, *F* (1, 24) = 4.60, *MSE* = 880.91, *p*<.05, η_p_^2^ = .16. The responses in the NP condition (643 ms) were significantly longer than those in the control condition (631 ms). The two-way interaction was not significant, *F*<1, indicating that the 15 ms of the NP effect in the distractor-first condition (647 ms vs. 632 ms) was statistically comparable to the 10 ms of the NP effect in the target-first condition (640 ms vs. 630 ms). No significant results were found in the error rate data.

#### Comparing the degree of distinctiveness

Experiments 1 and 2 differed in terms of the distinctiveness of the target and distractor letters. In Experiment 1, the distractor letters were distinctive in the sense that their color (i.e., red) was different from that of the other stimuli (i.e., black). In contrast, the distractor letters and all non-target stimuli were of the same color in Experiment 2. This difference is assumed to lead to different degrees of distinctiveness. Several findings support this assumption.

First, the mean response time in Experiment 1 (1050 ms, *SE* = 24.23) was significantly longer than that in Experiment 2 (637 ms, *SE* = 21.67), *F* (1, 43) = 161.44, *MSE* = 11740, *p*<.0001, η_p_^2^ = .79. Second, the mean error rate in Experiment 1 (37.5%, SE = 0.014) was significantly higher than that in Experiment 2 (10%, *SE* = 0.016), *F* (1, 43) = 161.66, *MSE* = 0.021, *p*<.001, η_p_^2^ = .79. Third, in Experiment 1, 74.21% (*SE* = 0.033) of the error responses incorrectly identified the target as the distractor letter, which was significantly more than the 54% (*SE* = 0.029) in Experiment 2, *F* (1, 43) = 21.14, *MSE* = 0.021, *p*<.0001, η_p_^2^ = .33. Taken together, these results indicate that the participants responded more slowly, committed more errors, and misidentified targets because of the presence of more distractor letters in Experiment 1 than in Experiment 2. These findings indicate that the distinctive target letter in Experiment 2 induces fewer distractions than the nondistinctive target in Experiment 1.

#### Comparing the degree of NP

The magnitudes of NP between the two experiments were compared to examine whether NP under RSVP decreases as target distinctiveness increases. A 2 (priming condition: NP vs. control)×2 (prime trial sequence: distractor-first vs. target-first)×2 (degree of target distinctiveness: nondistinctive in Experiment 1 vs. distinctive in Experiment 2) ANOVA revealed a significant priming condition×degree of distraction interaction, *F* (1, 43) = 6.05, *MSE* = 5230.40, *p*<.005, η_p_^2^ = .12. This interaction indicates that the 66 ms of NP in Experiment 1 (1083 ms vs. 1017 ms) was significantly stronger than that in the 12 ms of NP in Experiment 2.

## Discussion

The objective of the present study was to examine whether NP can be induced by the temporally presented stimuli. The results from the two experiments showed that (a) responses to a target were slower when the target served as a distractor in a previous display than when it did not, demonstrating NP under RSVP; (b) NP under RSVP was found regardless of whether the distractors appeared before or after the targets; and (c) NP under RSVP was stronger when the distractor was more distinctive. These findings provide clear evidence of the existence of NP under RSVP. The following section discusses how the extant accounts of NP explain the current findings.

### Implications for NP

The discussion focuses mainly on the four major accounts of NP: selective inhibition [1], [2], [11], [42], episodic retrieval [12], [13], feature mismatching [15], [16], and temporal discrimination [17]. This discussion does not seek to provide a critical evaluation of these accounts because the present research was not designed to test these accounts as competing explanations. I discuss how these accounts should be modified given the new empirical constraints from the present research to explain NP under RSVP.

#### Selective Inhibition

This account posits that NP occurs because the abstract mental representation of a distractor was actively inhibited in the prime trial and, consequently, the same item needs more activation to be identified in the subsequent probe trial [1], [2], [11], [22]. The current findings add three new insights into the inhibition of temporally presented distractors. First, the current findings suggest that the mental representation inhibition starts very quickly because all temporally presented stimuli are presented in a very short duration (e.g., 100 ms in the present research). This result is consistent with recent AB research on the fate of the distractors in RSVP [43], [44].

Second, the current findings are complementary to the findings reported by Lavie and Fox [45], offering additional support to the load theory of selective attention proposed by Lavie and colleague [45], [46]. This theory distinguishes two selective attention mechanisms, namely a perceptual attention mechanism and a cognitive control mechanism:

“a perceptual selection mechanism serving to reduce distractor perception in situation of high perceptual load that exhaust perceptual capacity in processing relevant stimuli and a cognitive control mechanism that reduces interference from perceived distractors as long as cognitive control functions are available to maintain current priorities” ([46], p. 339)

Lavie and Fox [45] tested the perceptual selection mechanism using an NP paradigm. Consistent with the first half story of the load theory, they found that an increase in perceptual load reduces NP. These findings were also interpreted to be consistent with the selective inhibition account of NP.

The findings of comparing the results between Experiments 1 and 2 from the present research are consistent with the cognitive control mechanism (i.e., the second half) of the load theory. Specifically, the key difference between Experiments 1 and 2 lies in the distinctiveness of the key distractor (i.e. in red in Experiment 1). The pairing of the two experiments is similar to that in standard attentional capture experiments in RSVP observed by Folk, Leber, and Egeth [47], which showed that when a distractor is distinctive, the distractor's feature will capture attention when this feature is relevant to the target selection (i.e., identity in this case). This attentional capture triggers AB-like effects that interfere with target processing. In fact, the current finding of a 30.9% error rates in the prime trials in Experiment 1 is comparable with the attentional capture effect in RSVP [47]. Most important, as shown in Folk et al. [47], this capture effect involves a high level of top-down attention control and does not involve low-level processing such as masking.

Integrating the involvement of a high level attention control of attention capture in the findings of the present research with the logic of the cognitive control mechanism of the selective attention mechanism of the load theory implies the following interpretation. The distinctive distractors in Experiment 1 (vs. Experiment 2) trigger (a) more mental resources for distractor inhibition, thus inducing strong NP, and (b) fewer mental resources for target processing, thus resulting in more errors and longer response time to targets (i.e., the attention capture effect). In other words, the reason for the increase in inhibition is presumably that distinctive distractors increase the selection difficulty, and consequently, the attention system poses stronger inhibitions to facilitate target selection [48]–[49].

Third, the inhibition account may not be straightforward in explaining why pre-target inhibition is comparable with post-target inhibition, as revealed by the statistically comparable NP under RSVP between conditions with the distractors presented before the targets and those presented after the targets. All post-target distractors in Experiments 1 and 2 were presented with one intervening item after the target, which is exactly the period in which the strongest AB occurs [6]–[8], [50]–[52]. This effect has been referred to as Lag 1 sparing [53]–[54], which has also been regarded as a period in which the target is undergoing a consolidation process from consciously unavailable state to a consciously available working memory [7], [55]. The significant NP found for distractors in this period suggests that the attention system attempts to prevent the consolidation process from interference with post-target distractors. Therefore, the inhibition of post-target distractors facilitates the consolidation.

This interpretation leads to a question. The Lag 1 sparing period is supposed to be with the lowest level of available mental resources for additional processing of one item after a target. Some researchers even consider that the consolidation process involves a central bottleneck that there are no resources for additional processing [7], [55]. Given that mental resources are likely required for distractor inhibition [44], the inhibition account, therefore, naturally predicts that the post-target distractor inhibition is significantly weaker than the pre-target distractor inhibition. However, this prediction is not supported by the current findings.

How can these findings be interpreted under the inhibition framework? Although the current findings showed a trend consistent with this prediction (i.e., 73 ms vs. 60 ms for pre- and post-target inhibition, respectively, in Experiment 1, and 15 ms vs. 10 ms for pre- and post-target inhibition, in Experiment 2, respectively), the differences were not statistically significant. Thus, it is possible that the non-supportive findings were due to the present research having insufficient statistical power to detect the differences. This possibility might not be very possible because the effect size of such suppression is expected to be large, given that the post-target suppression during AB has generally been recognized to be very strong. Another possibility is that the mental resources inhibiting the distractors during AB are different from the mental resources inhibiting the distractors that cause NP. Research has shown that different types of mental resources might be responsible for inhibition [10], [56]. Finally, it is also possible that consolidation per se does not occupy the whole bandwidth of the central bottleneck. Instead, consolidation plus inhibiting distractors consume all the bandwidth. The current research is not designed to test the above possibilities. These three possibilities will be a good topic for future research for testing the inhibition account of NP.

#### Episodic Retrieval

This account explains NP as a result of the automatic retrieval of “not-to-respond” information encoded along with a distractor in a prime trial in conflict with the current responding requirement to the target in the current probe trial [12]–[14], [57]. The findings in the present study add two constraints to the encoding aspects of the episodic retrieval accounts.

First, the current research implies that encoding the association between a distractor and the “not-to-respond” information should be done in a very short period of time such as within 100 ms. This constraint is not very salient in the previous research on spatial NP because the distractors in the spatial NP tasks are usually presented along with the targets until the participants respond to the target, which takes several hundreds of ms. Unlike the spatial NP procedure, all the distractors were presented very rapidly with successive masking by the other items.

Second, the current research implies that the degree of very rapid episodic encoding of the distractor information increases as the distinctiveness of the distractors increases. The distinctive items are encoded better than the less distinctive items [58]–[61], and these distinctiveness effects mainly concern encoding the target information without a strong time pressure. Taken together, the new insights gained from the present study on the episodic account is that this account has to assume that episodic encoding on the distractor “not-to-respond” and on the distinctiveness information occurs rapidly at a rate of 100 ms per item.

The episodic account is more straightforward in explaining why pre-target primes and post-target primes exhibited comparable effects. In RSVP, people not only have to deliberately ignore pre-target distractors, but also post-target distractors that are presented within a period in which the target is being processed because these post-target distractors may interfere with the target processing. Therefore, the “not-to-respond” tags are likely to be assigned to pre-target distractors and post-target distractors that are presented close to the targets. Accordingly, comparable NP is expected to be observed to both kinds of distractors because of equivalence in resolving the conflict associated with the “not-to-respond” association.

#### Feature Mismatching

This account explains that because the color of a distractor in a prime trial is different from the color of the item in the subsequent probe trial, this mismatch of features of the same item induces a further checking process, resulting in NP [15], [16]. A straightforward prediction derived from this account is that the degree of NP increases as the degree of mismatch increases, and that a comparable degree of NP is expected when the degree of mismatch is comparable. This account seems to have difficulty explaining the difference of NP under RSVP between Experiments 1 and 2 because of the comparable degree of feature mismatch in the two experiments. The feature mismatching account has to further assume that the mismatching checking process is asymmetrical, with a distinctive-to-nondistinctive mismatch triggering a longer checking than a nondistinctive-to-distinctive mismatch, to provide a satisfactory explanation on this difference.

#### Temporal Discrimination

Milliken et al. [17] explains NP as the result of the attention system taking a longer time to categorize a probe target as “new” or “old” when it was a prime distractor than when it was not. This categorization seeks to ascertain whether an item should be processed by a quicker memory-based route (i.e., when it is an old one) or by a slower algorithm-based route [62], [63]. The categorization time is assumed to vary as a function between the quality of match with the current target and previous items. This results in NP because the system needs extra processing of the current target as “new” when it matches with a previous distractor to a certain degree. Similar to the feature mismatching account, the temporal discrimination account predicts comparable NP when the degree of the match between the prime distractor and probe target is comparable. It is also difficult to explain why NP was found to be stronger in Experiment 1 than in Experiment 2 because the black-to-red conditions and the red-to-black conditions seem to have a comparable match.

Can these findings be explained by the asymmetrical discriminability between the distinctive-to-nondistinctive condition and the nondistinctive-to-distinctive condition? This is not likely. If the degree of distinctiveness increases the encoding of association between an item and its features, as mentioned in the episodic retrieval account above, the association between a distractor and “new” is expected to be stronger when it is a distinctive distractor than when it is not. Thus, the categorization time should be shorter, resulting in weaker NP in distinctive-to-nondistinctive conditions (i.e., in Experiment 1) than in nondistinctive-to-distinctive conditions (i.e., in Experiment 2). This notion contradicts the findings of the current research.

### Further Remarks on Explaining NP

It is important to note that the current research does not seek to test the different explanations of NP as mentioned above. All suggested difficulties and constraints of a particular account do not necessarily discount that the mechanism described by this account contribute to NP under RSVP. Different mechanisms may be operated complementarily. In fact, NP is likely caused by more than one mechanism [3], [64]–[66].

In the comparison between Experiments 1 and 2, there was a confounding between distractor distinctiveness and task difficulty. The distractor is less distinctive and the task is less difficult in Experiment 2 than in Experiment 1. NP may have been weaker in Experiment 2 because people could respond very quickly before any interference occurred [67]–[68]. This slowing hypothesis helps explain why NP was often not found without probe distractors (i.e., because responses can be made very quickly when there are no probe distractors). Experiment 2 was originally designed to test the occurrence of NP in an extreme condition in which the distractor identity was not encouraged to be processed. Experiment 2 was not designed to facilitate a fair examination of the effect of distractor distinctiveness on NP under RSVP. Although the NP difference between Experiments 1 and 2 was discussed in terms of distractor distinctiveness in the previous sections, the slowing hypothesis may also offer a straightforward and parsimonious explanation. Further research is needed to expound on these two factors.

### Conclusion

The results from the two experiments show that the participants require a longer processing time to identify a target that is a previous (temporally presented) distractor than when it is not a distractor. This NP under RSVP appears robust, as indicated by its occurrence in a pure feature search task in which the identities of the distractors are not encouraged to be processed (i.e., in Experiment 2). NP under RSVP is stronger when distractor distinctiveness is high than when it is low. There is no evidence that the NP for pre-target distractors is different from the NP for post-target distractors.

These findings provide several new empirical constraints to the current accounts of NP. First, these findings cannot be explained by the feature mismatch and the temporal discrimination accounts because the two accounts predict comparable effects between the NP in the distinctive-to-nondistinctive (red-to-black) conditions and that in the nondistinctive-to-distinctive (black-to-read) conditions. Second, to satisfactorily explain the current findings, the selective inhibition account needs to be assume further that (a) mental representation inhibition starts very quickly because all temporally presented stimuli are presented within in a very short duration (e.g., 100 ms in the present study), (b) the distinctive distractors require more mental resources for distractor inhibition, thus inducing strong NP, and (c) the attention system attempts to prevent the post-target consolidation process from interference by inhibiting post-target distractors. Third, to satisfactorily explain the current findings, the episodic retrieval account need to assume further that (a) encoding the association between a distractor and the “not-to-respond” information should be done within a very short period of time such as within 100 ms, (b) the degree of very rapid episodic encoding of distractor information increases as the distinctiveness of the distractors increases, and (c) the episodic information on “not-to-respond” is assigned to post-target distractors when the distractors are temporally close to the target.
